# Health-Promoting of Polysaccharides Extracted from *Ganoderma lucidum*

**DOI:** 10.3390/nu13082725

**Published:** 2021-08-07

**Authors:** Ewa Seweryn, Anna Ziała, Andrzej Gamian

**Affiliations:** 1 Department of Medical Biochemistry, Wroclaw Medical University, 50-368 Wrocław, Poland; ewa.seweryn@umed.wroc.pl; 2 Hirszfeld Institute of Immunology and Experimental Therapy, Polish Academy of Sciences, 53-114 Wrocław, Poland; andrzej.gamian@hirszfeld.pl

**Keywords:** *Ganoderma lucidum*, phytochemicals, natural medicines, health-promoting properties, immunomodulation, antidiabetic, antioxidation, antineurodegeneration

## Abstract

Medicinal mushrooms are rich sources of pharmacologically active compounds. One of the mushrooms commonly used in traditional Chinese medicine is *Ganoderma lucidum* (Leyss. Ex Fr.) Karst. In Asian countries it is treated as a nutraceutical, whose regular consumption provides vitality and improves health. *Ganoderma lucidum* is an important source of biologically active compounds. The pharmacologically active fraction of polysaccharides has antioxidant, immunomodulatory, antineurodegenerative and antidiabetic activities. In this review, we summarize the activity of *Ganoderma lucidum* polysaccharides (GLP).

## 1. Introduction

**Figure 1:**
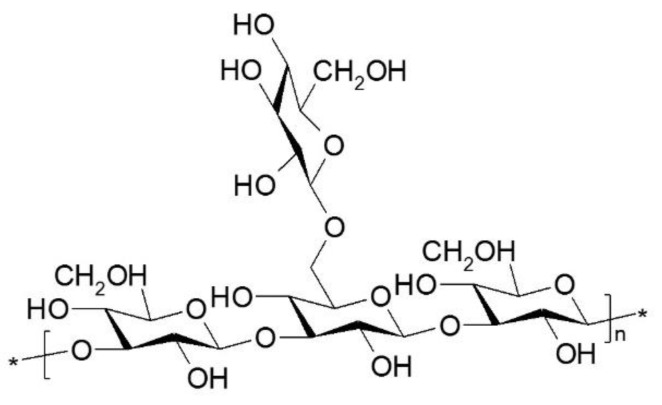
Bioactivity of polysaccharides and triterpenoids from *Ganoderma lucidum*.

**Figure 2:**
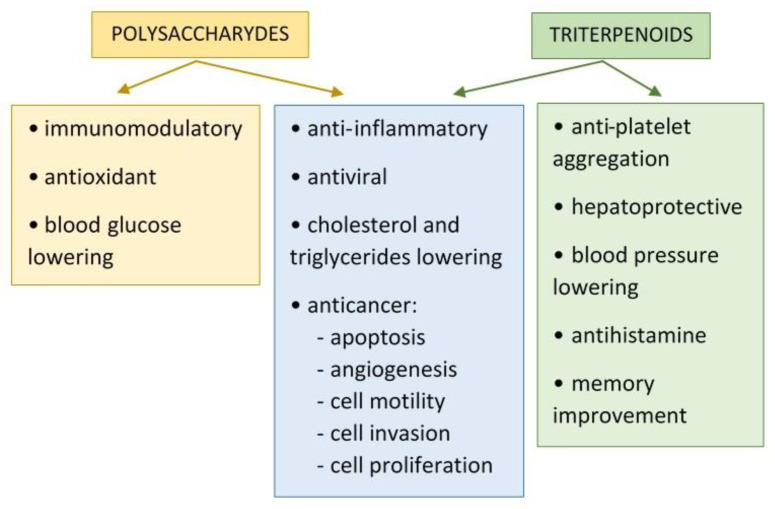
Structure of β-1,3-glucan with β-1,6-branching.

Nutraceuticals or functional food are terms used interchangeably to refer to products that provide health benefits and improve the quality of life. Such substances, in addition to nutritional value, contain bioactive components with a broad spectrum of activity. In traditional Chinese medicine essences and extracts made from medicinal mushrooms are used. The beneficial properties of medicinal mushrooms are associated with a wide range of bioactive compounds present in the fruiting bodies, mycelium and spores [1]. One of the most intriguing medicinal mushrooms is the Ganoderma lucidum (Lingzhi, Reishi), which has been used in East Asia for centuries. Potions from this mushroom have been used in traditional Chinese medicine for years to improve health, increase vitality and extend life. *G. lucidum* is a source of bioactive compounds, such as polysaccharides, triterpenes, proteins, steroids, nucleotides, glycoproteins, peptides, sterols, fatty acids and trace elements [2,3]. To demonstrate the numerous pharmacological effects of the above components, a study was carried out using molecular techniques and animal models. The great potential of *G. lucidum* in supporting the treatment of the present diseases of civilization, such as diabetes, neurodegenerative diseases, atherosclerosis or inflammation, has been demonstrated. Polysaccharides obtained from *Ganoderma lucidum* show a wide range of pharmacological antioxidant, immunomodulatory, antineurodegenerative, antidiabetic, anti-inflammatory, anticancer and antibacterial properties (Figure 1) [4,5]. The polysaccharide is a type of natural polymer made of monosaccharides linked by α and β glycosidic bonds to form main and side chains. The core of the chemical structure of polysaccharides is β-glucan with various β glycosidic bonds and at its branching points are β-(1→6). Moreover, polysaccharides can be heteroglucans, with a mixture of α-(1→3) glycosidic bonds of glucan, β-(1→6) mannan, β-(1→4) galactan or contain protein components (Figure 2) [6].

Polysaccharide chains can form triple helices that are stabilized by hydrogen bonds. The occurrence of such a tertiary triple helix structure was demonstrated in β-D-glucans from various fungi, as well as from *G. lucidum* [7]. The bioactivity of such β-D-glucans is determined by their β structure (Figure 2). The chemical structures of polysaccharides vary depending on the method of isolation and the type of culture broth used. Apart from pure β-glucans, isolated mainly from *G. lucidum* fruiting bodies, there are also heterofucans, heteromannans and their complexes with peptides [8]. The best known polysaccharides from *G. lucidum*, the pure glucose polymer (GLP-1) and the polymer built of β-D-glucose and α-D-galactose (GLP-2) show particular antioxidant activities [9]. These properties result from the presence of various functional groups that are electron and hydrogen donors [8,10]. Modifications to polysaccharides are now commonly performed to improve the structural composition, bonding, molecular weight and ionic nature of such a polymer. By changing these parameters, you can also change their physicochemical properties and biological functions [11]. Chemically modified polysaccharides from *G. lucidum* as a result of acetylation, carboxymethylation, alkylation or sulfonylation are proven in vitro tests and on cell lines and animals [12,13,14]. As a result of acetylation or carboxymethylation of a polysaccharide, the exposure of hydroxyl groups changes, which results in better solubility of the modified polysaccharides and increasing its antioxidant activity in vitro. The sulphate derivative of polysaccharides, in turn, has a strong anti-inflammatory effect, as it interferes with the action of proinflammatory cytokines and inhibits the activation of complement in in vivo studies [14]. It can be definitely stated that the antioxidant effect of polysaccharides increases with the help of chemical modifications, which is a tool for producing preparations based on natural polysaccharides. Studies of recent years indicate that polysaccharides are the most pharmacologically active and health promoting compo-nents [15,16]. In this review, we show the latest experimental (in vivo and in vitro) ap-proaches to clarify the biological properties of polysaccharides and the possibility of using them as a nutraceuticals (Table 1).

## 2. Bioactivity of Polysaccharides from *Ganoderma lucidum*

### 2.1. Antioxidative Activity

Under physiological conditions, there is a balance between the formation of reactive oxygen species and their elimination by the free radical scavenging system. Excessive levels of ROS cause redox imbalances and lead to oxidative damage to the tissues. Protein, lipid and DNA damage caused by oxidative stress and the resulting elevated levels of reactive oxygen species (ROS) are important factors in the onset and development of diseases. Medicinal fungi such as *Ganoderma lucidum* have antioxidant and prooxidative properties, which is used in combination therapy for some diseases [17,37]. Polysaccharides isolated from *G. lucidum* showing antioxidant activity protect tis-sues against ROS toxicity and also help maintain the oxidative status of the body [46,47]. The Chinese Food and Drug Administration (CFDA) has approved a drug based on a polysaccharide extracted from spores of *G. lucidum* [37]. This preparation is only used in China for polymyositis, dermatitis and muscular dystrophy [37]. It is also one of the few non-hormonal drugs used in the treatment of refractory myopathy and in combination therapy with glucocorticoids [48]. According to the conducted in vivo experiments, polysaccharides from *G. lucidum* show anti-inflammatory and protective effects against oxidative stress in the heart, liver, spleen and skeletal muscles [49]. GLPs induce the synthesis of superoxide dismutase (SOD), glutathione peroxidase (GPx), catalase (CAT), glutathione S-transferase (GST), mitochondrial succinate dehydrogenase (SDH) and reduce glutathione, which protects the endothelium of blood vessels [17]. They however reduce the activity of nitric oxide synthase (NOS), cytochrome P450, xanthine oxidase and myeloperoxidase, which significantly affect the dysfunction of the vascular endothelium and induce atherosclerosis [18,50]. Undoubtedly, oxidative stress plays a significant role in the etiology of many metabolic diseases that disrupt the proper functioning of many organs. Prolonged oxidative stress leads to the aging of the body and the occurrence of many age-related diseases [50,51]. In vivo studies have shown that GLP have a beneficial antioxidant effect as a result of a decrease in lipid peroxidation levels and an increase in the activity of antioxidant enzymes [19,50]. Depending on the GLP dose used, the above antioxidant properties were checked in mice exposed to ɣ irradiation and cervical carcinoma in rats [52,53]. Further in vivo studies demonstrated that low molecular mass polysaccharides (GLP-1 and GLP-2) show a proportionally higher antioxidant and immunomodulatory activity [21]. Both polysaccharides were administered to mice with immunosuppression caused by cyclophosphamide administration [21]. Polysaccharides increased the number of white blood cells and lymphocytes, which meant positive effect on hematopoiesis. Serum IgG and IgA immunoglobulin levels were also tested and elevated IgA was found [21]. This study demonstrated the immunomodulatory activity of polysaccharides naturally occurring in medicinal mushroom. Other studies have shown the effect of two polysaccharides, GLP and GLP_UD_, on the activity of superoxide dismutase and glutathione peroxidase and malondialdehyde level in the serum and liver of mice fed a high fat diet [54]. Polysaccharides were heteropolysaccharides, consisting mainly of glucose and significantly lower amounts of fructose, mannose, galactose, xylose, rhamnose, glucuronic acid and galacturonic acid. Mice administered with *G. lucidum* extracted polysaccharides for 30 days showed increased activity of antioxidant enzymes, in proportion to the dose of GLP used. Moreover, malondialdehyde levels decreased [54]. In many biochemical transformations oxygen and nitrogen free radicals are created, which are extremely reactive and cause mitochondrial dysfunction. The long-term effects of oxidative stress significantly accelerate the aging processes and is related to numerous neurodegenerative diseases, metabolic syndromes and neoplasms. However, supplementation with polysaccharide preparations from *G. lucidum* could contribute to the improvement of our lives, which should be the goal of further searches. Further studies should focus on the assessment of the degree of toxicity of polysaccharide preparations and the assessment of the potential of their efficacy in clinical trials. So far, the use of the polysaccharide from *G. lucidum* as a drug has only been approved by the CFDA.

### 2.2. Immunomodulatory Activity

**Figure 3:**
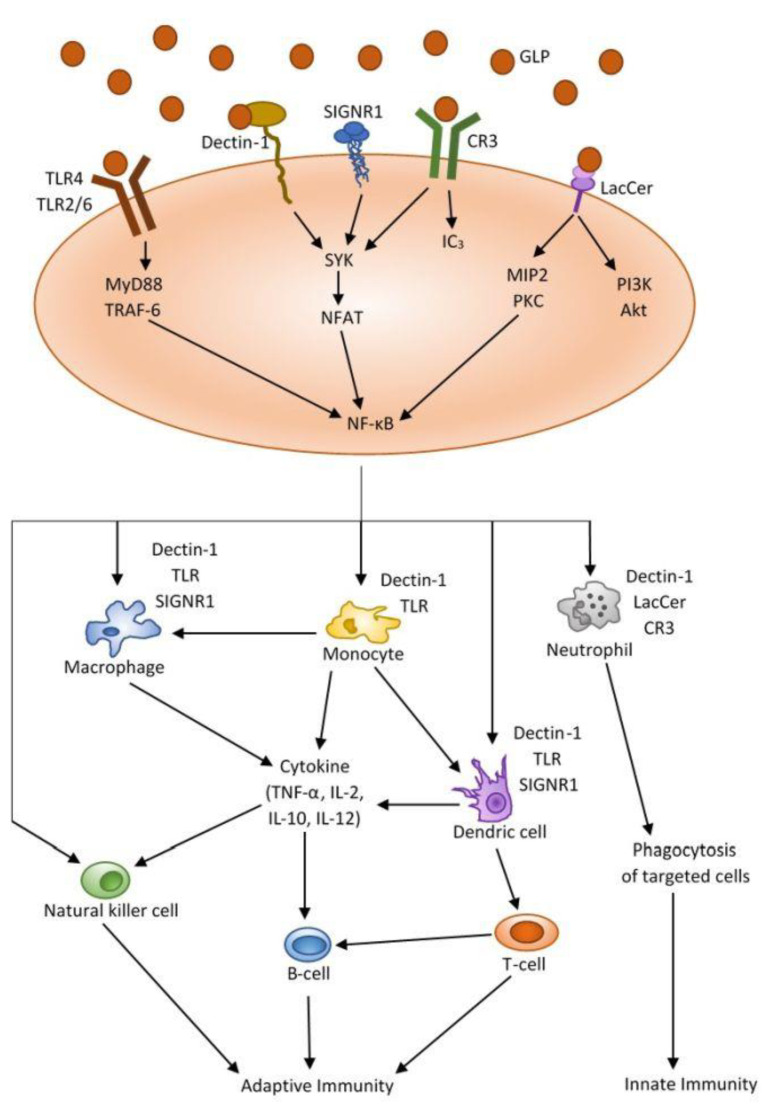
Mechanisms of immune activation by polysaccharides from *Ganoderma lu-cidum*. (Akt—protein kinase B; CD25—α-chain of interleukin-2 receptor; CD71—trans-ferrin receptor-1; CR3—complement receptor-3; DAG—diacylglycerol; IP3—inositol triphosphate; LacCer—lactosylceramide receptor; MIP2—macrophage inflammatory protein-2; MyD88—myeloid differentiation primary response 88; NFAT—nuclear factor of activated T-cells; NO—nitric oxide; PKA—protein kinase A; PKC—protein kinase C; SIGNR1—C-type lectin receptor; SYK—tyrosine protein kinase; TRAF-6—TNF-receptor associated factor 6).

Current biochemical and clinical studies have shown that polysaccharides from *Ganoderma lucidum* are potent immunomodulators. The immunomodulatory activity of polysaccharides is associated with their influence on effector cells, such as macrophages, B and T lymphocytes, Natural Killer cells (NK cells) and dendritic cells [22,55,56]. GLP’s have been found to increase T and B cell proliferation through Toll-like receptor 4 (TLR4). As a result of interactions with TLR4/TLR2 receptors, signal induction occurs through a p38 mitogen-activated protein kinase (p38 MAPK) (Figure 3) [23,57].

Lin at al. has shown that GLP also induces the activation and maturation of human dendritic cells derived from monocytes, through NF-ĸB nuclear factor (nuclear factor κ-light-chain-enhancer of activated B cells) signaling and MAPK protein kinases [24]. Moreover, GLP has been shown to enhance the function of chemotaxis and phagocytosis of neutrophils [24]. These processes involve the phosphorylation of tyrosine kinases, p38 MAPK, Src (proto-oncogene tyrosine-protein kinase Src), PI3K (phosphatidylinositol 3-kinase) and protein kinase C [24]. Cell-wall polysaccharides isolated from *G. lucidum* have also been shown to induce innate immune cytokines, tumor necrosis factor-α (TNF-α), interferon γ (IFN-ɣ) and interleukin-2 (IL-2) in human peripheral blood mononuclear cells (PRMC) [25,58].

Macrophages are responsible for the phagocytosis of pathogens, reacting to chemokines that induce their recruitment to the site of tissue damage. Serine-threonine kinases (Akt1 and Akt2) play the most important role in regulating macrophage activation [59]. Depending on the signaling cascades, activation stages and stimuli of the cellular environment, macrophage polarization can be defined as M1 or M2 [60]. Classically activated macrophages (M1) produce pro-inflammatory and cytotoxic molecules, such as reactive oxygen species (ROS), nitric oxide, TNF-α, IL-1β and IL-6, and chemokines [61]. The rFIP-glu polysaccharide from *G. lucidum* has a dose dependent modulating effect on the activation of RAW264.7 macrophages [26]. These results show that the rFIP-glu recombinant polysaccharide transcriptionally regulates the expression of inflammatory mediators, TNF-α, NO, arginase II, IL-1 and IL-6, in LPS-stimulated macrophages (Figure 3) [26]. rFIP-glu strongly promotes polarization of M1 macrophages by initiating proinflammatory reactions, while lowering IL-10, a marker of M2 macrophages. The authors suggest that rFIP-glu polysaccharide may affect the conversion of M2 to M1, which plays a key role in the treatment of many diseases. A study by Mallard et al. found synergistic effects of β-glucans derived from three different medicinal mushrooms on human macrophages [27]. These preparations, also derived from *G. lucidum*, induced an immunostimulatory response leading to the expression of proinflammatory mediators, such as Il-1, IL-6 and TNF-α [26,27]. At the same time, they reduced the expression of the anti-inflammatory cytokine IL-10. This is the first research on synergistic immunomodulatory effects of natural preparations from medicinal mushrooms. Therefore, the results of the above study provide mechanistic insight into the bioactivity of such complex preparations, as well as their application in the design of bioactive drugs. Based on current scientific research, it can be concluded that *Ganoderma lucidum* is a promising source of nutraceuticals with broad-spectrum drug therapeutic potential. Due to the huge number of biologically active compounds, it is a potential source of natural medicinal substances with low toxicity. However, further pharmacokinetic and clinical studies is required to de-termine the toxicity of these compounds.

### 2.3. Antineurodegenerative Activity

**Figure 4:**
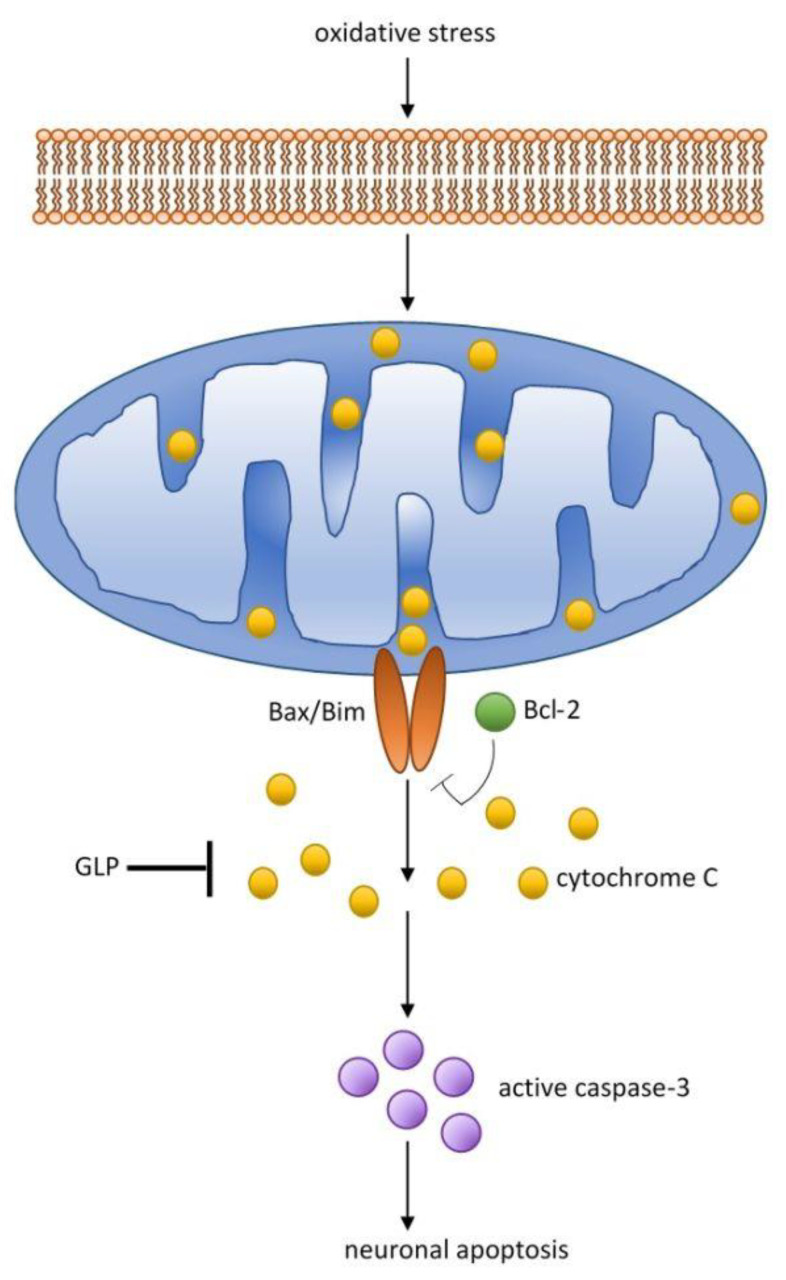
Possible mechanisms of neuronal damage in Alzheimer’s disease by accumu-lation Aβ and releasing proinflammatory cytokines (Aβ—β-amyloid; AD—Alzheimer’s disease; BACE1—β- secretase; Fc—Fc receptors; RAGE—complement receptors advanced glycation and products; SRs—scavenger receptors).

Redox imbalance in cells, as well as excessive or unregulated production of inflammatory mediators, is an element of the induction of neurodegenerative diseases, such as atherosclerosis, diabetes, Alzheimer’s disease, Parkinson’s disease and Huntington’s disease [28,29,62]. In neurodegenerative diseases, activated microglia is observed, which releases proinflammatory and anti-inflammatory cytokines and neurotoxic mediators. In a study on the LPS-induced inflammation microglia cell line, treated with GLP for two hours, the expression of proinflammatory cytokines IL-1 and IL-6 and induced NO synthase (iNO) was inhibited [30]. Further study results showed that GLP is also a potent inhibitor of amyloid β (Aβ) stimulated primary mouse microglia (Aβ), which may be indicative of a modulation of neurological inflammation. GLP also significantly increased the expression of the anti-inflammatory cytokine TGFβ in both the microglia cell line (BV2) and the primary microglia cell line [30]. Based on the above in vitro studies, it can be assumed that GLP may act in early stages Alzheimer’s disease to reduce inflammation. In a similar study, the effect of *G. lucidum* fruiting body extract (GLE), which contained a large polysaccharide fraction, was tested on BV2 cell line [63]. GLE decline the level of pro-inflammatory cytokines in cells by modulating the signaling pathways of NF-ĸB and MAP kinases that regulate the synthesis of proteins involved in inflammatory processes [63]. It is therefore highly probable that *G. lucidum* fruiting body extract can also play a role in the prevention of neurodegenerative diseases by modulating signaling pathways. Recent studies have shown that polysaccharides from *G. lucidum* has neuroprotective effects and impairs neurotoxicity induced by β amyloid peptide [31]. Further reports found that administration of GLP to rats protects their hippocampus against oxidative damage (Figure 4) [32,64]. These data initiated the possibility of polysaccharide in the treatment of Alzheimer’s disease.

**Figure 5:**
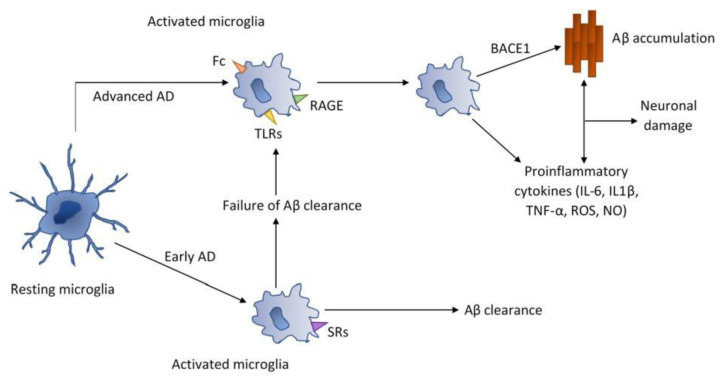
Elementary mechanisms of protective effect of polysaccharides from *Ganoderma lucidum*. GLP arrest neuronal apoptosis through increase expression of Bcl-2 and decrease expression of caspase-3, Bax and Bim (Bax—Bcl-2-like protein 4; Bcl-2—B-cell lymphoma 2 protein (family of regulator proteins); Bim—Bcl-like protein 11).

Activated microglia releases pro- and anti-inflammatory cytokines and cytotoxic mediators [65]. Microglia can remove dead neurons as a result of phagocytosis but also cause death of live neurons by phagoptosis [66]. Phagoptosis is involved in the loss of neurons during neurodegeneration of the brain. Microglia acts as the main immune defense in the central nervous system (CNS). In the active phase, phagocytic microglia migrate and accumulates at the site of injury. Microglia is the brain macrophage and has the ability to remove apoptotic cells (Figure 5) [66,67,68]. Polysaccharides extract from *G. lucidum* significantly reduced amyloid-induced neurotoxicity [31]. Further work demonstrated the *G. lucidum* spore effect on rat hippocampus, which protected against oxidative damage [32]. Another significant findings was that GLP is capable of inhibiting microglial activation in rats with Parkinson’s disease [33]. Other important process underlying aging and age-related diseases is the gene methylation cycle. Measurements of genome hypomethylation and hypermethylation of specific genes is emerging in understanding the aging processes [34]. In this direction, a study was carried out on the effects of alcohol extracts of triterpenes and polysaccharides from *G. lucidum* on the regulation of DNA methylation in rats with induced aging [34]. Aging was induced by intraperitoneal administration of D-galactose for eight weeks [69]. Elevated DNA methyltransferase levels and improved morphology of hippocampal pyramidal cells were found in brain tissues after treatment with alcohol extracts from *G. lucidum* [34]. Histochemical results have shown that the extracts used can have a positive effect on neuronal apoptosis and brain atrophy, and reduce the expression of the Alzheimer’s marker, β-amyloid (Aβ1-42) [34]. Based on the above study, it can be concluded that alcohol extracts from *G. lucidum* can regulate DNA methylation which affects the progression of Alzheimer’s disease. Further work into DNA methylation will help to elucidate the mechanism behind these processes.

### 2.4. Antidiabetic Activity

The long-term effects of diabetes reveal the dysfunction and failure of many organs [20]. Many experiments have shown that animals with induced diabetes have a higher level of oxidative stress and redox imbalance is closely related to disease development [35]. The organism has its own antioxidant system that should maintain a balance between ROS formation and the free radical scavenging system. From animal studies with streptozotocin-induced diabetes, it was found that the antioxidant, both enzymatic and nonenzymatic systems were significantly impaired [35,70,71]. This concerned the activity of free radical scavenging enzymes, SOD, GPx, CAT and oxidative stress. The polysaccharides from *G. lucidum* acted as exogenous antioxidants and restored the endogenous redox balance by reducing malondialdehyde levels and inducing the expression of antioxidant enzymes [35,36,70,71]. Studies of ultrastructural changes in pancreatic β cells also confirmed damage to these cells in a group of animals with induced oxidative stress and diabetes [35]. External oxidative stress significantly affected the redox balance and led to damage to the mitochondria. However, after applying the polysaccharide (GLPs) to a group of animals, the ultrastructure of the mitochondria of the pancreatic islet cells was maintained by restoring the redox balance [35]. Excessive production of ROS in cells damages the mitochondrial membrane, oxidizes proteins, introduces mutations into DNA, which ultimately causes mitochondrial dysfunction [20,72]. Most of the endogenous ROS are of mitochondrial origin and indirectly contribute to the development of insulin resistance [73,74]. Natural extract of the polysaccharide from *G. lucidum* also reduced insulin resistance and damage to pancreatic islet cells, and successfully reversed the entire process of diabetes development along with the prolonged duration of action [35]. The above studies suggest that in future clinical trials, which are lacking regarding polysaccharides from *G. lucidum*, the level of oxidative stress, the dose of the polysaccharide and the duration of its action on the given tissue should be taken into account. Oxidative stress, which occurs even in the early stages and at physiological glucose levels, plays a key role. Recent studies in rats with induced diabetes have shown that GLP supplementation reduces inflammation and leads to an increase in the beneficial intestinal microflora that protects the organism against infections [45]. These results provide further insight into the beneficial effects of the polysaccharide from *G. lucidum* on the regulation of metabolism and modulation of the intestinal dysbiosis [38]. Nonenzymatic reactions of glucose with proteins or lipids lead to the formation of advanced glycation end products (AGE) that disturb the organism’s biochemical and physiological functions. Current therapies have limited effectiveness, tolerance and significant side effects. Therefore, interest in natural therapies are increasing. The most interesting findings was that *G. lucidum* polysaccharides are safe and as effective as antioxidants [39,40].

Another important finding was that polysaccharides have a antihyperglycemic effect [40,41]. To this end, water extracts from *G. lucidum* were also tested, reflecting normal, daily consumption of mushrooms. It was found that the administration of water extracts to laboratory animals with induced diabetes significantly reduced blood glucose levels [42,75,76]. The hypoglycemic effect of polysaccharides have been extensively studied in vitro and in vivo [77,78,79]. Hypoglycemic effect of polysaccharides from *G. lucidum* was found in rats with streptozotocin induced diabetes [42,43]. GLP also had the ability to relieve morphotic changes in the kidneys and reduce oxidative stress. In this study, GLP was found to cause the hypolipidemic effects, significantly reducing total cholesterol and triglycerides [43]. The polysaccharide from *G. lucidum* administered to rats with insulin resistance improved vascular endothelial dysfunction [44]. There was a decrease in the levels of hydrogen peroxide, triglycerides and total cholesterol, which significantly improved the vascular endothelium [44].

Genetic and environmental conditions as well as bacterial microflora play an im-portant role in diabetes. A strong relationship between diabetes mellitus and intestinal dysbiosis has been reported in the literature [80,81]. Current research indicates that GLP has an effect on intestinal dysbiosis that has been associated with type 2 diabetes [45]. Intestinal dysbiosis mediates immune disorders, chronic inflammation and the development of diabetes mellitus through abnormal production of its metabolites. As a result of treatment with polysaccharide from *G. lucidum* for four weeks, the level of beneficial bacteria increased and the hyperglycemia and hyperlipidemia were alleviated in type 2 diabetic rats [45]. However, more research on this topic needs to be undertaken before the association between polysaccharides from *G. lucidum* and intestinal dysbiosis is more clearly understood. Bioactive polysaccharides represent new strategies for the treatment of disorders associated with metabolic diseases. After all, potential pharmacological approaches to circumvent the harmful effects of oxidative stress by reducing exogenous and endogenous sources of free radicals, inhibiting the inflammation induced by them and including the use of antioxidant polysaccharides from *G. lucidum* are extremely promising.

## 3. Conclusions

Treatment with natural substances has been used for thousands of years, but mainly in the Far East countries. In Chinese medicine, mycotherapy, treatment with preparations obtained from medicinal fungi, occupies a significant place among therapies. It involves the use of extracts and preparations obtained from mushrooms known in Traditional Chinese medicines. One of such fungi is *G. lucidum*, the properties of which are appreciated in Asian countries, where it is treated as a nutraceutical. It is believed that the regular administration of *G. lucidum* extracts provides vitality, supports the treatment of many diseases and even extends life. The research results indicate that polysaccharides from *G. lucidum* may be helpful in the conventional therapy of many diseases, due to their pharmacological properties. In depth research into the mechanisms of action GLP could lead to the development of effective chemopreventive agents of natural origin. However, supplementation with natural extracts also carries a potential risk of interaction with the administered drugs. Our recognition of the advantages of mushrooms as a healthy food and source of biologically active substances are constantly developing. However, despite promising published data, further research is required to assess the therapeutic benefits of medicinal mushrooms. The investigation of treatment with nutraceuticals without side effects is a challenge, which may improve a strategy for the treatment metabolic diseases.

## Figures and Tables

**Table 1:** Biological activities, mechanism of action/effects of polysaccharides from *Ganoderma lucidum*.

Biological Activities	Bioactive Components/ Extracts	Origin	Mechanism of Action/ Effects	References
Antioxidative activity	GLP_S_ GLP_UD_	Fruit body	Induce synthesis of SOD, GPx, CAT, GST, SDH, GSH in serum. Decrease in MDA. Reduce hyperlipidemia.	[17]
	GLP_S_	Spore	Reduce activity of NOS, cyt. p450, xanthine oxidase, myeloperoxidase. Decrease in MDA, ROS in endothelial cells.	[18]
	GLP_S_	Fruit body	Anti-hydroxyl free radical and anti-superoxide radical ability. Decrease in lipid peroxidation. Increase activity of SOD, CAT, GPx.	[19]
	GLP_S_	Fruit body	Induce synthesis of SOD, CAT; Reduce lipid peroxidation; Decrease inflammatory cytokine: TNF-α, IL-6, IL-1β; Scavenging abilities on DPPH.	[20]
Immunomodulatory activity	GLP_S_	Fruit body	Promote hematopoiesis. Increase in IgA levels. Regulate of IgG levels.	[21]
	GLP-Au	Fruit body	Induce of DC maturation; Increase in T and B cells proliferation. Enhancement of NK cells, IL-2 and IFN-γ. Increase in IL-6, IL-12, IL-1β, TNF-α, IFN-ɣ.	[22]
	GLP_S_	Fruit body	Inactivation of MAPK and Akt/mTOR signaling pathways.	[23]
	GLP_S_	Fruit body	Activation of dendritic cells; Induce of IFN-ɣ, IL-10 in human DC. Activation of NF-ĸB, p38MAPK, ERK1/2 pathways during DC maturation.	[24]
	α-glucan β-glucan	Fruit body Mycelium	Increase in TNF-α, IFN-γ, IL-2 in human PBMC. Increase in IL-2, IFN-ɣ in Th1 cells. Induce of IL-17 in PBMC.	[25]
	rFIP-glu	Fruit body	Activation of RAW264,7 macrophages. Regulate expression of TNF-α, NO, IL-1, IL-6. Induce phosphorylation of Akt.	[26]
	β-glucan	Fruit body	Regulate expression of IL-1, IL-6, TNF-α in human macrophages. Reduce expression of IL-10.	[27]
	GLP_S_	Fruit body	Regulate expression of IL-1, IL-6, iNOS in microglial cells. Increase expression of TGFβ. Modulation of microglial morphology and phagocytosis.	[28]
	GLE	Fruit body	Modulation NF-ĸB and MAPK signaling pathway. Inhibition of pro-inflammatory cytokines. Inhibition of nitrate in microglial cells.	[29]
Antineurodegenerative activity	GLE	Fruit body	Neuroprotective effect. Reduce amyloid toxicity. Decrease neurotoxicity. Decrease phosphorylation of JNK, c-Jun and p38MAPK.	[30,31]
	GLE	Spore	Neuroprotective effect in hippocampus. Increase GSH, Gpx. Decrease MDA. Increase ATP and CytQx.	[32]
	GLP_S_	Fruit body	Protection dopaminergic neurons against inflammation. Inhibition of microglial activation. Decrease TNF-α, IL-1β.	[33]
	GLE	Fruit body	Regulation of DNA methylation. Improve morphology of hippocampal pyramidal cells. Reduce of β-amyloid.	[34]
Antioxidative activity Antidiabetic activity	GLP_S_	Fruit body	Significant anti-hydroxyl free radical and anti-superoxide radical activity. Recover SOD activity. Restoring the redox balance. Alleviate insulin resistance.	[35]
	GLP_S_	Fruit body	Reduce blood glucose level. Decrease LDH, HbA1c, total cholesterol, triglycerides. Reduce expression of TNF-α, IL-1β	[36]
Anti-inflammation activity	GLP_S_	Spore	Effective on polymyositis, dermatomyositis and muscular dystrophy.	[37]
	GLP_S_	Spore	Reduce serum levels of TNF-α, IL-1β, MCP-1, IL-6. Decrease LDL. Modulation of the intestinal dysbiosis.	[38,39]
Antihyperglycemic activity	GLE	Fruit body	Decrease serum glucose and insulin levels. Increase activity of SOD, CAT, GPx, GSH. Decrease lipid peroxidation.	[40,41,42]
	GLP_S_	Fruit body	Reduce blood glucose and HbA1c levels. Increase activity of SOD, CAT. Reduce serum AGE. Reduce total cholesterol and triglycerides.	[43]
	GLE (β-D-glucan)	Fruit body	Improve vascular endothelial dysfunction. Increase circulating endothelial cells. Decrease hydrogen peroxide; Decrease triglycerides and cholesterol.	[44]
Antioxidative activity Anti-inflammation activity	GLP_S_	Fruit body	Decrease IL-1β, IL-6. Decrease triglycerides, total cholesterol. Increase CAT, SOD, GPx. Reduce gut microbiota dysbiosis.	[45]

## Data Availability

Not applicable.
